# Lynch-related adenocarcinoma in a cervical gastric inlet patch

**DOI:** 10.1055/a-2566-1388

**Published:** 2025-04-09

**Authors:** Veronique Van der Voort, Olivier Plomteux, Gauthier Demolin, Noella Blétard, Raf Bisschops, Jérémie Jacques, Philippe Leclercq

**Affiliations:** 1 Department of Gastroenterology and Hepatology, Centre Hospitalier Universitaire (CHU) de Limoges, Limoges, France; 2 Department of Gastroenterology and Hepatology, Clinique CHC MontLégia, Liège, Belgium; 3 Department of Oncology, Clinique CHC MontLégia, Liège, Belgium; 4 Department of Pathology, Clinique CHC MontLégia, Liège, Belgium; 5 60182 Department of Gastroenterology and Hepatology, University Hospitals Leuven, Leuven, Belgium; 6 Department of Gastroenterology and Endoscopy, Centre Hospitalier Universitaire (CHU) de Limoges, Limoges, France

A 65-year-old asymptomatic man with MSH2-related Lynch syndrome underwent routine upper endoscopy. A 13-mm Paris Is sessile lesion, suspected of malignancy, was found in the middle of a cervical gastric inlet patch (GIP) ( Video 1 ; Fig. 1 **a** ). The lesion was removed en bloc by endoscopic submucosal dissection (ESD), using a clip-with-line traction technique ( Fig. 1 **b-d** ). Histology revealed a well-differentiated adenocarcinoma pT1a m2 arising from ectopic gastric metapasia, with free resection margins and no presence of prognostic risk factors for lymph node metastasis ( Fig. 2 ). Loss of expression of MSH2 and MSH6 was found, and *Helicobacter pylori* was absent in the stomach and inlet patch. Surveillance endoscopy at 3 and 12 months showed no local recurrence or metachronous lesions.

Endoscopic submucosal dissection of an early-stage adenocarcinoma that developed from gastric metaplasia in a cervical gastric inlet patch.Video 1

**Fig. 1:**
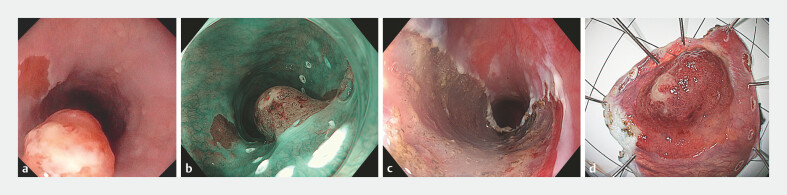
Endoscopic findings of early adenocarcinoma arising from ectopic gastric mucosa in the cervical esophagus in a 65-year-old man with Lynch syndrome. **a** White light shows the sessile lesion with ulcerated surface. Next to the lesion another inlet patch can be seen. **b** Narrow-band imaging (NBI) shows the irregular vascular pattern of the lesion. Some discrete bleeding and ulceration can also be noticed. The white dots are the delineation of the area to be resected by endoscopic submucosal dissection (ESD) involving the entire inlet patch. **c** Resection ulcer in the proximal esophagus after ESD. **d** Resection specimen with a lesion of 13 × 10 mm within a recognizable inlet patch.

**Fig. 2:**
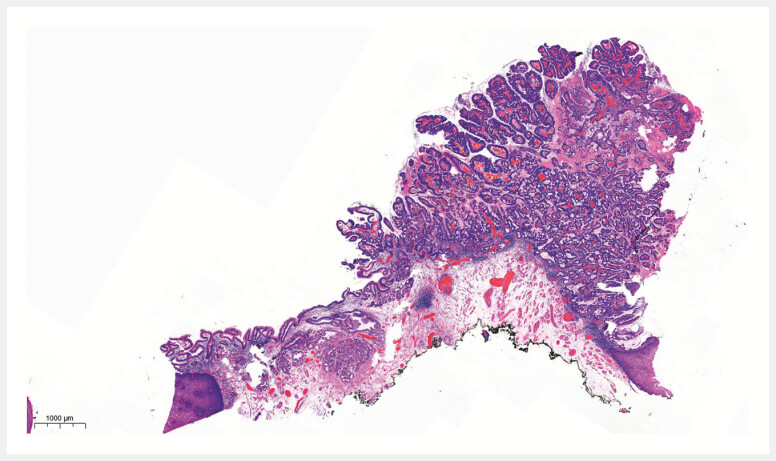
Histological image of the pT1a m2 adenocarcinoma of the proximal esophagus, arising from ectopic gastric mucosa.

Lynch syndrome is one of the most common hereditary cancer predisposition syndromes, significantly increasing the risk of colonic and extracolonic cancers 1 . Gastric and duodenal cancer has incidences of up to 13% and 11%, respectively, with a higher incidence of gastric cancer in MSH2 mutation carriers 1 2 . If diagnosed in time, early carcinoma can be successfully treated with ESD, although symptoms often appear late or not at all. Nevertheless, the routine screening of Lynch syndrome patients via esophagogastroduodenoscopy remains debated, with varying guideline recommendations 3 4 5 . Additionally, the absence of *H. pylori* indicates that this is not the only mechanism promoting the development of gastric cancer in Lynch patients, despite *H. pylori* screening often being the only routine recommendation for screening in multiple guidelines 3 4 5 . This case endorses the importance of meticulous endoscopic assessment of not only the stomach and duodenum but also the proximal esophagus, which harbors an underestimated risk when a GIP is present. “Don’t skip the GIP!”

Endoscopy_UCTN_Code_CCL_1AB_2AC_3AB

## Notes

Endoscopy E-Videos https://eref.thieme.de/e-videos
*E-Videos* is an open access online section of the journal *Endoscopy* , reporting on interesting cases and new techniques in gastroenterological endoscopy. All papers include a high-quality video and are published with a Creative Commons CC-BY license. Endoscopy E-Videos qualify for HINARI discounts and waivers and eligibility is automatically checked during the submission process. We grant 100% waivers to articles whose corresponding authors are based in Group A countries and 50% waivers to those who are based in Group B countries as classified by Research4Life (see: https://www.research4life.org/access/eligibility/ ). This section has its own submission website at https://mc.manuscriptcentral.com/e-videos .

## References

[1] Kumar S Farha N Burke CA Upper Gastrointestinal cancer surveillance in Lynch Syndrome Cancers 2022 14 1000 10.3390/cancers14041000 35205747 PMC8869779

[2] Aarnio M Sankila R Pukkala E Cancer risk in mutation carriers of DNA-mismatch-repair genes Int J Cancer 1999 81 214 218 10.1002/(sici)1097-0215(19990412)81:2<214::aid-ijc8>3.0.co;2-l 10188721

[3] Van Leerdam ME Roos VH van Hooft JE Endoscopic management of Lynch syndrome and of familial risk of colorectal cancer: European Society of Gastrointestinal Endoscopy (ESGE) Guideline Endoscopy 2019 51 1082 1093 10.1055/a-1016-4977 31597170

[4] Stjepanovic N Moreira L Carneiro F Hereditary gastrointestinal cancers: ESMO Clinical Practice Guidelines for diagnosis, treatment and follow-up Ann Oncol 2019 30 1558 1571 10.1093/annonc/mdz233 31378807

[5] Monahan KJ Bradshaw N Dolwani S Guidelines for the management of hereditary colorectal cancer from the British Society of Gastroenterology (BSG)/Association of Coloproctology of Great Britain and Ireland (ACPGBI)/United Kingdom Cancer Genetics Group (UKCGG) Gut 2020 69 411 444 31780574 10.1136/gutjnl-2019-319915 PMC7034349

